# JOB EXPECTATIONS VS. REALITY: WORKPLACE EXPERIENCES FROM THE NURSING HOME FRONTLINES

**DOI:** 10.1093/geroni/igad104.3445

**Published:** 2023-12-21

**Authors:** Vivian Miller, Jennifer Wagner, Lauren Maziarz, Melissa Burek, Julia Bell, Kaley Perry

**Affiliations:** Bowling Green State University, Bowling Green, Ohio, United States; Bowling Green State University, Bowling Green, Ohio, United States; Bowling Green State University, Bowling Green, Ohio, United States; Bowling Green State University, Bowling Green, Ohio, United States; Bowling Green State University, Bowling Green, Ohio, United States; Bowling Green State University, Bowling Green, Ohio, United States

## Abstract

Nursing assistants have been at the frontlines of nursing home care, providing direct resident care and services to aging adults. The U.S. Bureau of Labor Statistics projects an increased employment need for nurse aides over the next ten years. However, the annual rate of nurse aide turnover is over 120 percent. To better understand the unique factors that contribute to nurse aide turnover in nursing homes and assisted livings, this sequential mixed-methodological study design reports findings of activities, relationships, and job interactions that contribute to nurse aide turnover. Guided by a socio-ecological model, qualitative findings from 17 nurse aides (N=17) reveal interpersonal factors, such as relationships with others at the workplace and relationships with residents, as impacting turnover. Institutional and Community factors were also found to impact turnover, including staffing levels, resources, education, and compensation. Informed by the interview findings, there were 239 nursing home or assisted living nurse aides (N=239) that participated in the quantitative portion of this study. Over one-quarter of nurse aides (27.80%) reveal that getting to know the residents was one of the best things about working in a nursing home or assisted living. Of direct care tasks, nurse aides reported physical injury or strain (20.16%), and the physical demands of the job (20.16%) as the greatest challenges. Taken together, findings from this study reveal that using community-engaged research methods in nursing home research may lead to a more nuanced understanding of the workplace needs and desires of nursing assistants.

